# 49. Schnitzler syndrome: a case of chronic urticaria and monoclonal gammopathy

**DOI:** 10.1093/rap/rky034.012

**Published:** 2018-09-20

**Authors:** Catherine M McGrath, Karen M J Douglas

**Affiliations:** 1 Rheumatology Research Group, University of Birmingham, Birmingham, UNITED KINGDOM; 2 Rheumatology, The Dudley Group NHS Foundation Trust, Dudley, UNITED KINGDOM

**Introduction:** A comprehensive medical history is essential in new patient appointments and is constructed from the building blocks of previous medical encounters. But in the same way that a house is built of bricks, a diagnostic conundrum may be solved by pulling strands of the medical history together to make a coherent whole as observed in a rare case of the auto-inflammatory condition Schnitzler syndrome.

**Case description:** A 44-year-old Caucasian woman was referred to the rheumatology department of a district general hospital for rather non-specific intermittent small joint arthralgia. She reported a progressive, persistent non-pruritic urticarial rash which was spreading from trunk to limbs and which she now felt affected her ability to deliver care as an allied healthcare professional. She disclosed years of drenching night sweats and maintained a slim but stable weight. She saw immunology for the urticarial rash, managed with fexofenadine and levocetirizine with limited benefit. She saw haematology for an IgM monoclonal gammopathy of undetermined significance (MGUS) and remained under their surveillance. In her family history, her grandmother had rheumatoid arthritis, her mother was treated for systemic lupus erythematosus and her son had a selective IgA deficiency. On examination, an extensive urticarial rash was noted over the patient's abdomen, chest and back. There were no findings of a classical inflammatory arthritis. There was a solitary, 1 cm non-painful lymph node palpable in the left axilla. The patient had mild abdominal tenderness and had been simultaneously referred also for investigations after complaining of breast pain. The patient had a working diagnosis of both MGUS and chronic urticaria but neither fully explained the night sweats or intermittent arthralgia and prompted widening the list of differential diagnoses which for chronic urticaria includes autoimmune urticaria, cholinergic urticaria, cold urticaria, mastocytosis, and periodic fever syndromes including Muckle-Wells syndrome. At the first rheumatology visit, the suspicion was of an (as yet unknown) unifying diagnosis and an internet search of the main symptoms brought up Schnitzler syndrome at the top of the list. A telephone discussion the following day with the National Amyloidosis Centre confirmed that the patient satisfied the Strasbourg criteria for Schnitzler syndrome and prompted an urgent referral. In the meantime, routine investigations were completed in line with the patient’s symptoms and history. A bilateral mammogram showed benign and fibrocystic change and enlarged lymph nodes in both axillae. A CT thorax, abdomen and pelvis showed mildly enlarged bilateral axillary nodes with no mediastinal or abdomino-pelvic adenopathy and with a normal liver and spleen. An US guided biopsy of an enlarged axillary lymph node showed non-specific benign reactive lymphadenopathy. Immunology showed a marginally raised rheumatoid factor but was negative for anti-nuclear antibody and extractable nuclear antigens, and ferritin, C3, C4 complement levels, C1 inhibitor, lactate dehydrogenase and immunoglobulin E values were all in normal ranges. Paraproteinaemia had been gradually increasing with immunoglobulin M (IgM) >15 g/L at presentation. There were moderately raised inflammatory markers. A punch biopsy of the skin of the mid back showed features consistent with an urticarial vasculitis without fibrinoid necrosis of blood vessels and slides were also sent for review by an international expert in the condition in Strasbourg. While awaiting skin biopsy results, the patient had her first visit to the national centre with a diagnosis of Schnitzler syndrome followed by prescription of the interleukin-1 antagonist anakinra with rapid improvement in urticarial rash and night sweats. The patient has gained weight and is back to being comfortable wearing short sleeved tops. She relies on daily anakinra to keep symptoms at bay and had re-occurrence of rash when she forgot her medication during a weekend away. An auto-inflammatory focused genetic screen showed no mutations. She remains under specialist care with a rapid and sustained drop in serum amyloid A protein levels and a slowing down in increase of IgM levels. She still has some persistent sweats.

**Discussion:** Schnitzler syndrome is a rare auto-inflammatory condition first described in 1972 with the index case dying at the age of 88 years. It likely arises from a defect in the innate immune system and manifests as chronic urticaria/IgM(G) gammopathy/arthralgia/raised inflammatory markers. At least 300 cases have been documented worldwide, mostly in adult Caucasians, but the number of identified cases is increasing with greater awareness of the condition. The family history is typically negative for symptoms of Schnitzler syndrome (one case report of a family positive for a monoclonal gammopathy) and there are no known risk factors for the condition. Currently genetic screening typically includes a panel of auto-inflammatory genes but no expanded and familial genome wide studies have been reported. The role of interleukin (IL)-1 matured in the inflammasome is important since a daily subcutaneous injection of the IL-1 receptor antagonist anakinra rapidly controls symptoms. The rapid response to anakinra helps confirm the condition but symptoms rapidly return when the drug is stopped. The course of the disease is generally benign but 15-20 % of patients develop a lymphoproliferative disorder prompting examination of lymph nodes and bone marrow and long-term surveillance.

**Key Learning Points:** Pattern recognition works not only when we already know the pattern but equally when there is strong clinical suspicion of one. Schnitzler syndrome is a rare auto-inflammatory condition that typically responds rapidly to IL-1 antagonists with significant improvement in quality of life. No known risk factors for Schnitzler syndrome are known but familial clustering of both auto-immune and auto-inflammatory conditions (as in this case) should be explored in more detail.

**Disclosure: C.M. McGrath:** Honoraria; Pfizer. Grants/research support; National Institute of Health Research. **K.M.J. Douglas:** None.

